# A carboxylesterase, Esterase-6, modulates sensory physiological and behavioral response dynamics to pheromone in *Drosophila*

**DOI:** 10.1186/1741-7007-10-56

**Published:** 2012-06-21

**Authors:** Thomas Chertemps, Adrien François, Nicolas Durand, Gloria Rosell, Teun Dekker, Philippe Lucas, Martine Maïbèche-Coisne

**Affiliations:** 1 Université Pierre et Marie Curie, UMR 1272, Physiologie de l'Insecte, Signalisation et Communication, F-75252, Paris, France; 2 INRA, UMR 1272, Physiologie de l'Insecte, Signalisation et Communication, F-78026, Versailles, France; 3 University of Barcelona, Faculty of Pharmacy, Unit of Medicinal Chemistry, 08028, Barcelona, Spain; 4 Swedish University of Agricultural Sciences, Department of Plant Protection Biology, 23053, Alnarp, Sweden

**Keywords:** carboxylesterase, esterase 6, olfaction, pheromone, signal termination

## Abstract

**Background:**

Insects respond to the spatial and temporal dynamics of a pheromone plume, which implies not only a strong response to 'odor on', but also to 'odor off'. This requires mechanisms geared toward a fast signal termination. Several mechanisms may contribute to signal termination, among which odorant-degrading enzymes. These enzymes putatively play a role in signal dynamics by a rapid inactivation of odorants in the vicinity of the sensory receptors, although direct *in vivo *experimental evidences are lacking. Here we verified the role of an extracellular carboxylesterase, esterase-6 (Est-6), in the sensory physiological and behavioral dynamics of *Drosophila melanogaster *response to its pheromone, *cis*-vaccenyl acetate (cVA). Est-6 was previously linked to post-mating effects in the reproductive system of females. As Est-6 is also known to hydrolyze cVA *in vitro *and is expressed in the main olfactory organ, the antenna, we tested here its role in olfaction as a putative odorant-degrading enzyme.

**Results:**

We first confirm that *Est-6 *is highly expressed in olfactory sensilla, including cVA-sensitive sensilla, and we show that expression is likely associated with non-neuronal cells. Our electrophysiological approaches show that the dynamics of olfactory receptor neuron (ORN) responses is strongly influenced by Est-6, as in *Est-6° *null mutants (lacking the *Est-6 *gene) cVA-sensitive ORN showed increased firing rate and prolonged activity in response to cVA. *Est-6° *mutant males had a lower threshold of behavioral response to cVA, as revealed by the analysis of two cVA-induced behaviors. In particular, mutant males exhibited a strong decrease of male-male courtship, in association with a delay in courtship initiation.

**Conclusions:**

Our study presents evidence that *Est-6 *plays a role in the physiological and behavioral dynamics of sex pheromone response in *Drosophila *males and supports a role of *Est-6 *as an odorant-degrading enzyme (ODE) in male antennae. Our results also expand the role of *Est-6 *in *Drosophila *biology, from reproduction to olfaction, and highlight the role of ODEs in insect olfaction.

## Background

A sense that lacks spatial resolution requires a high temporal resolution for accurate location of signal sources in space. Insect pheromone responses exemplify this, with a capability of resolving and responding to pheromone filaments in 100 to 200 ms [1]. For such a system to operate it requires not only fast responses to 'odor on', but also to 'odor off'. Odor-off responses imply inactivation of odorant signals. Several mechanisms have been proposed to participate in signal cessation or reduction within insect olfactory hairs (or sensilla), involving either olfactory receptors (Ors) or molecules interacting with them (reviewed in [2]). In *Drosophila melanogaster*, when Or genes were expressed in another olfactory receptor neuron (ORN) than their native ORN by using the 'empty neuron' system (*Δhalo *mutant), signal termination was similar to what had been observed in their native ORN, suggesting that Ors play a key role in signal dynamics [3]. However, when an Or from the silk moth *Bombyx mori *was expressed in *Drosophila *T1 sensilla, termination of the response was rapid, whereas it was delayed when this receptor was expressed in another type of sensilla than the T1s, suggesting that the cellular environment of Ors could also play a role in the dynamics of the response [4]. In particular, fast degradation of odorants in the vicinity of Ors by odorant-degrading enzymes (ODEs) has been proposed as a mechanism contributing to the termination of ORN responses. Pheromone degradation *in vitro *by antennal extracts ([5]; reviewed in [6,7]), by purified antennal enzymes [8,9], as well as enzymatic inhibition *in vivo *(reviewed in [10,11]) strongly support this hypothesis. Various enzyme families were described as candidate ODEs, such as carboxylesterases, aldehyde oxidases, epoxide hydrolases, glutathione-S-transferases or cytochrome P450 (reviewed in [7]). Few ODEs have been both identified at the molecular level and functionally characterized *in vitro*. Among them, carboxylesterases involved in pheromone/odorant degradation were the most studied [8,9,12-14]. However, involvement of ODEs in odorant processing has never been directly demonstrated *in vivo*.

In *Drosophila melanogaster*, the molecular mechanisms involved in the reception of a male-produced olfactory pheromone, *cis*-vaccenyl acetate (cVA), are intensely studied. In males, cVA suppresses male-male courtship [15] and promotes male-male aggression [16]. cVA also suppresses courtship towards recently-mated females, as cVA is transferred to the female with the seminal fluid [17]. Or67d, an Or mediating the sensory and behavioral responses to the cVA [15,16,18,19] is expressed in T1 trichoid sensilla [18]. LUSH, an odorant-binding protein (OBP), and SNMP1, a putative membrane bound coreceptor, are also required for cVA sensitivity [15,18,20-22]. Elements of binding and reception of cVA within T1 sensilla are thus well documented, but the mechanisms of cVA inactivation are unknown. In our search for putative factors that are involved in cVA degradation we noted that an extracellular carboxylesterase (EC 3.1.1.1), Esterase-6 (Est-6; CG6917), which is transferred during copulation to the female with the seminal fluid [23], hydrolyzes cVA *in vitro *[24]. Interestingly in males, *Est-6 *is not only expressed in the ejaculatory duct [25], but also in the antennae [26-29], suggesting that Est-6 could play a role in pheromone processing.

In the present work we determined *in vivo *the role of *Est-6 *in cVA olfaction. We studied the phenotypes of several *Est-6 *mutant and control strains at the electrophysiological and behavioral levels. Our results demonstrate that *Est-6 *enables flies to detect and respond to the temporal dynamics of cVA stimulation. In addition, cVA-triggered behaviors are also modified in mutants, suggesting that *Est-6 *is of behavioral significance.

## Results

### *EST-6 *is highly and broadly expressed in male antennae

**Figure 1:**
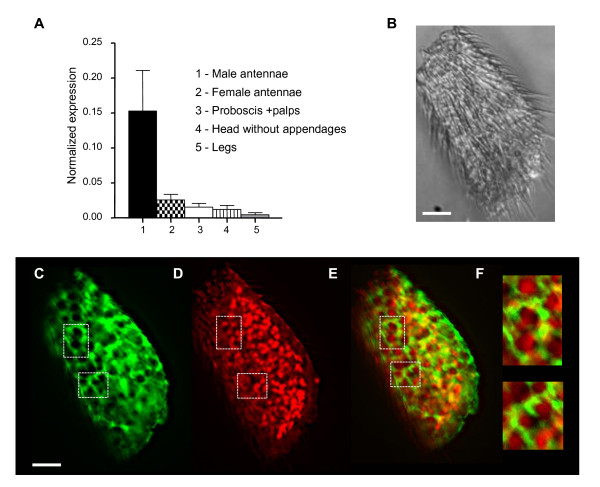
***Est-6 *expression in antennae**. **(A) **Quantitative PCR (qPCR) analysis of *Est-6 *expression on cDNAs from wild-type (*CS*) male and female antennae, male gustatory appendages and heads without sensory appendages. *Est-6 *expression level was normalized to that of *pgk*. Data were obtained from triplicate biological experiments and are given as the mean ± SEM. **(B) **Third antennal segment, posterior view of left antenna. Medial is to the left. **(C) **Expression of *Est-6 *in *Est-6*^Gal4^/*UAS-mCD8-GFP *male. **(D) **Anti-ELAV staining of *Est-6*^Gal4^/*UAS-mCD8-GFP *antennae. **(E) **Merge of (C, D). **(F) **higher magnification of (E). Scale bars = 20 μM (except for (F), scale bar = 8 μm).

First we quantified the transcript levels between different chemosensory appendages by quantitative PCR (qPCR). *Est-6 *levels were ninefold higher in antennae compared to the proboscis (gustatory organ)/maxillary palps (olfactory organ) (Figure 1A). *Est-6 *was barely detectable in legs, which bear gustatory sensilla, including sensilla responding to female-specific pheromones [30]. Interestingly, we also observed a clear sexual dimorphism, as male antennae expressed 6.5-fold more *Est-6 *than the female antennae. To examine the expression pattern of *Est-6 *within chemosensory organs, we observed *Est-6-Gal4/UAS-mCD8-GFP *male antennae, which express green fluorescent protein (GFP) under the control of *Est-6 *promoter. *GFP *was widely expressed throughout the third antennal segment (Figure 1B, C). Moreover, most of the GFP^+ ^cells did not seem to coexpress ELAV, a neuronal marker (Figure 1D, F). *Est-6 *is thus highly and broadly expressed in male antenna. At the cellular level, a neuronal expression could not be completely excluded, but expression is mostly observed in olfactory accessory cells surrounding ORNs.

### ORN responses to cVA depend on *Est-6 *expression

**Figure 2:**
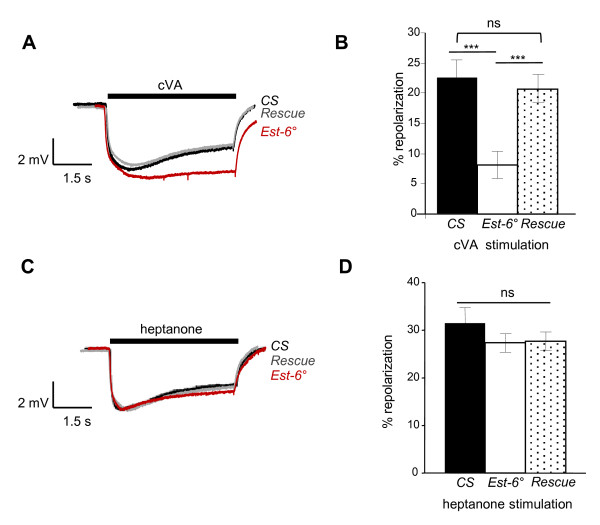
**Antennal responses of males to *cis*-vaccenyl acetate (cVA) and heptanone**. **(A) **Average electroantennography (EAG) plots from wild-type (*CS*, in black), null mutant (*Est-6°*, in red) and rescue (*Rescue*, in grey) strains during a 5-s stimulation with cVA. The horizontal bar indicates the duration of stimulus delivery. **(B) **Percentage of repolarization from the same genotypes calculated at the end of a 5-s stimulation with cVA. Mean ± SEM; *N *≥ 10 for each genotype and test. **(C) **Average EAG plots from *CS*, *Est-6° *and *Rescue *male flies during a 5-s stimulation with heptanone. **(D) **Percentage of repolarization from the same genotypes calculated at the end of a 5-s stimulation with heptanone. Mean ± SEM; N = 13 for each test. Student's t test, *P < 0.05; **P < 0.01; ***P < 0.001.

To test whether the olfactory response to cVA is modified in *Est-6° *males, we first recorded the responses of antennae by electroantennography (EAG). A dose response curve to cVA was established (Additional file 1, Figure S1). We selected a dose of cVA (200 μg/cartridge) that induced a high response with our system and performed long stimulation (5 s) in order to mimic an overstimulation of the antennae. In these conditions, the dynamics of EAG responses clearly differed between the *null *mutant *Est-6°*, which completely lacks *Est-6*, and the two control strains, that is, the wild-type strain *Canton S *('*CS*') and the rescue strain ('*Rescue*'), in which *Est-6 *expression was restored (Figure 2A): the depolarization was similar in the three strains but the repolarization was slower in *Est-6° *males. The repolarization rates at the end of the stimulation (Figure 2B) were reduced in Est-6° mutant compared to the controls (8% in *Est-6° *vs 22.4% in *CS *and 20.7% in the rescue strain). EAG results thus indicate that the lack of *Est-6 *in mutant flies affects the temporal dynamics of antennal responses to cVA, with a delayed signal termination. To test whether the *Est-6 *mutation affects the general functioning of the antennae, we measured their responses to a different odorant. We selected 2-heptanone, because this ketone is detected by a basiconic type sensillum that is distinct from cVA-responding trichoid sensilla [31] and because its chemical structure prevents degradation by esterases. In contrast to cVA, high doses of 2-heptanone did not elicit different responses between control and *Est-6° *males (Figure 2C, D), suggesting that the *Est-6 *mutation does not have a general effect on the olfactory detection.

**Figure 3:**
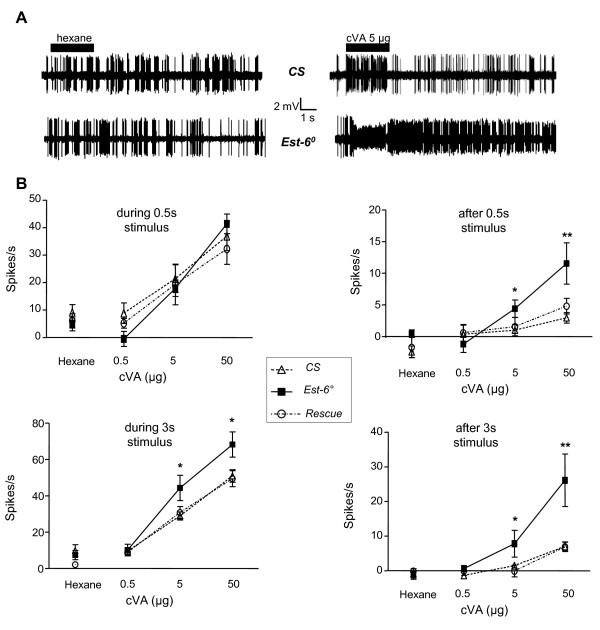
**T1 sensillum responses to *cis*-vaccenyl acetate (cVA) measured by single-sensillum recordings**. **(A) **Action potentials recorded from *CS *and *Est-6° *T1 sensilla stimulated for 3 s with solvent (left) or cVA (right). Horizontal bars indicate the duration of stimulus delivery. **(B) **Dose-response curves for T1 sensilla from *CS*, *Est-6° *and *Rescue *male flies: spike frequencies during the stimulation (0.5 s and 3 s), as well as for 9 s following the stimulus were calculated relatively to the prestimulus frequency. Mean ± SEM; N ≥ 8 for each data point. Student's t test, *P < 0.05; **P < 0.01.

As the relationship between EAGs and response characteristics of underlying sensory neuron is tenuous, we therefore verified the temporal response characteristics of individual ORNs in T1 sensilla, which are specifically tuned to cVA, using single-sensillum recordings (SSR). We used physiological doses of cVA and brief stimulations as well as higher doses and long stimulations. Both firing rate and response duration of T1 neurons upon stimulations with 5 and 50 μg of cVA were affected in *Est-6° *mutants compared to *CS *and *Rescue *flies (Figure 3A, B). With 0.5-s stimulation and both doses of cVA, *Est-6° *mutants exhibited a delayed signal termination, as revealed by the increased spiking rate after stimulation (4.4 spikes/s in *Est-6° *vs 0.9 in *CS *and 1.0 in *Rescue *flies for a dose of 5 μg; 13.5 spikes/s in *Est-6° *vs 2.9 in *CS *and 4.7 in *Rescue *flies for a dose of 50 μg). With prolonged, 3-s stimulations, at low and high doses of cVA, both firing rate and duration of the response were significantly increased in mutant males compared to *CS *and *Rescue *strains (Figure 3B). In summary, following cVA stimulation, T1 sensilla in *Est-6° *males responded with a delayed signal termination, whatever the dose and the duration of the stimulation. In addition, firing rate during stimulation was also increased in response to prolonged stimulations.

### *Est-6 *modulates courtship behavior

**Figure 4:**
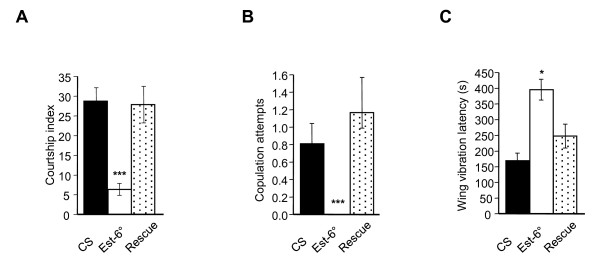
**Analysis of male-male courtship**. The courtship index (CI) is the fraction of time spent in courtship activity in the 10-minute observation period. **(A) **CI of males of the indicated genotypes paired with a decapitated *CS *target male. **(B) **Number of copulation attempts. **(C) **Wing vibration latency in seconds. Mean ± SEM; N ≥ 30 for each genotype and test. Mann Whitney, *P < 0.05; ***P < 0.001.

To examine whether *Est-6 *mutation could influence cVA-induced behaviors, we first measured male-male sexual behavior by measuring the courtship index (CI) toward a *CS *target male. Under daylight conditions and when paired with an immobilized male, a wild-type male courts typically with a CI of 10% [15]. This percentage increased significantly when visual cues were suppressed (CI of 25 to 28% in control strains, Figure 4A). The CI was however strongly reduced in *Est-6° *(6.5%). Similarly, copulation attempts were absent in *Est-6° *mutants (Figure 4B), indicating a decrease in courtship vigor. Conversely, the latency of wing vibration was higher in the mutant strain (Figure 4C), indicating a delay in courtship initiation. In the rescue strain, all these phenotypes were restored. Absence of *Est-6 *thus correlated with a decreased male-male courtship, suggesting that the mutation enhances the antiaphrodisiac effect of cVA.

**Figure 5:**
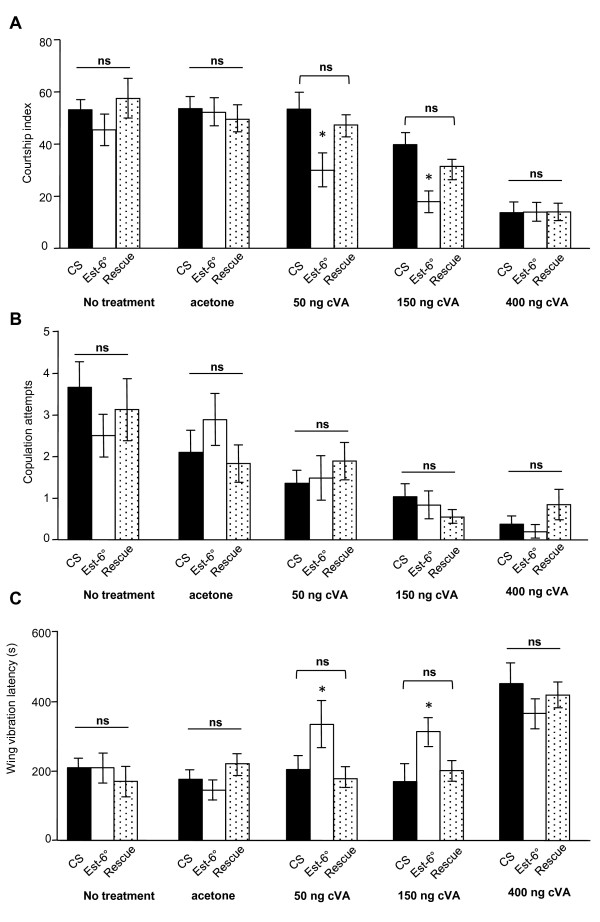
**Analysis of male-female courtship**. **(A) **CI of males of the indicated genotypes paired with a decapitated *CS *target virgin female. Females were treated either with acetone, 50, 150 or 400 ng of synthetic *cis*-vaccenyl acetate (cVA). **(B) **Number of copulation attempts. **(C) **Wing vibration latency in seconds. Mean ± SEM; N ≥ 30 for each genotype and test. Mann Whitney, *P < 0.05.

Courtship of males is thought to be inhibited by cVA [17,32], however, male gustatory pheromones such as *Z*-7-tricosene are also antiaphrodisiac for males [33]. To evaluate whether the behavioral modification observed in *Est-6 *mutant flies could be directly linked to cVA, we analyzed the heterosexual courtship of males. Under dim red light, the CI of *CS *and *Rescue *males to *CS *decapitated virgin females ranged between 53.2% to 57.6%, respectively, and did not significantly differ from those of *Est-6° *mutant males (45.4%) (Figure 5A). Copulation attempts (Figure 5B) and courtship initiation (Figure 5C) was also comparable in the three strains. Absence of *Est-6 *had thus no effect on heterosexual courtship, suggesting that the perception of female pheromones is not affected. As heterosexual courtship is driven by gustatory and olfactory cues, this result suggested that *Est-6 *mutation did not interfere with their detection and integration.

We subsequently scored the CI of males to *CS *virgin females treated with exogenous cVA or solvent only. With solvent only, the CI was comparable in *CS*, *Rescue *and *Est-6° *males (53.4%, 50.8% and 52.8%, respectively; Figure 5A) and reached similar level as without any treatment. With the highest dose of cVA, the CI of control and *Est-6° *flies dropped to around 14%. The intermediate dose of 150 ng (approximately two-thirds male equivalent) induced a decrease of courtship in the three genotypes, however this decrease was more pronounced in *Est-6 *(19.2%) than in *CS *(39.8%) and *Rescue *(32,2%) males. This decrease was associated with a delay in courtship initiation, as shown by the corresponding wing vibration latency (Figure 5C). More interestingly, with the lowest dose (50 ng, 1/4 male equivalent) the CI of *CS *and Rescue males was unaffected, whereas it was significantly reduced in *Est-6° *males (30.4%), with again a delay in courtship initiation. A decrease in copulation attempts was observed when females were treated with cVA, but this decrease was comparable in the three strains (Figure 5B). The antiaphrodisiac effect of cVA was thus dose-dependent in the three strains. However, the amount of synthetic cVA required to inhibit the male courtship was lower for *Est-6° *than for control males, suggesting that the mutants presented a lowest threshold of response to the pheromone.

### *Est-6 *modulates aggression-promoting behavior

**Figure 6:**
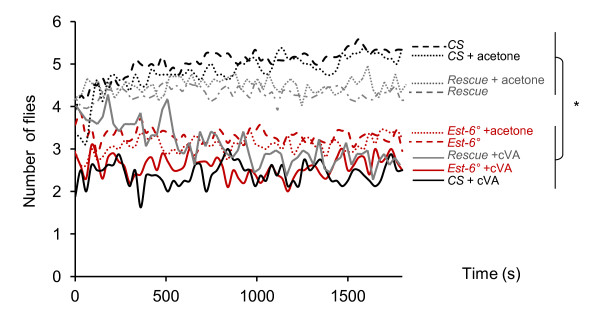
**Dispersal test of male flies from a food resource**. Number of flies of the indicated genotype on the food cup in the absence or presence of synthetic *cis*-vaccenyl acetate (cVA). Dispersion reflects the aggressiveness level under group-housing conditions. *CS*: dark dashed line; *CS *+ acetone: dark dotted line; *CS *+ cVA: dark bold line; *Rescue*: grey dashed line; *Rescue *+ acetone: grey dotted line; *Rescue *+ cVA: grey bold line; *Est-6°*: red dashed line; *Est-6° *+ acetone: red dotted line; *Est-6° *+ cVA: red bold line. N ≥ 8. Two-way analysis of variance (ANOVA) followed by Bonferroni post hoc test.

As cVA also promotes male-male aggression [16], we finally compared aggression behavior between *Est-6° *and control males. Male aggressiveness was analyzed indirectly, using a dispersal test. The dispersal of male flies competing for a food resource is indeed correlated with the level of aggression [16]. In the absence of synthetic cVA, control males quickly aggregated on the food resource and remained there for at least 30 minutes after introduction into the chamber (Figure 6). Solvent only (acetone) did not have any effect. In the presence of synthetic cVA at high dose (500 μg) after initial attraction to the resource, the number of *CS *and *Rescue *flies on the food cup declined, indicating aggression-induced dispersal. *Est-6° *males exhibited also increased dispersal, but this behavior was observed even in absence of synthetic cVA. As cVA is a volatile pheromone, its concentration is proportional to the number of male flies in a given environment. Our results suggest that the level of cVA emitted in the test chamber by six males was sufficient to trigger dispersal of *Est-6° *males, but not of control males.

### Olfactory behavior in response to food odors is not altered in mutant flies

The response of male flies to olfactory cues from food was evaluated using a trap assay [34]. Mutant flies were able to detect and locate the food paste equally well as control males (Additional file 2, Figure S2A). As the performance in this trap assay is a good reflect of the olfactory function [34], the result indicates that the lack of *Est-6 *in the antennae or the genital tract does not have a general effect on olfactory-driven behaviors in mutants. Finally, the locomotor activity of mutant males was also comparable to control males, which demonstrates that *Est-6 *mutation does not affect locomotor activity in general (Additional file 2, Figure S2B).

## Discussion

This study demonstrates that a carboxylesterase, Est-6, previously linked to post-mating effects in the reproductive system of *D. melanogaster *females, plays also a role in the sensitivity and dynamics of ORNs tuned to cVA, the volatile fruit fly pheromone. We also infer that this physiological function of Est-6 at the peripheral olfactory level is required for normal male behavioral responses to cVA.

Est-6 is known as an extracellular enzyme in the male genital tract [23] and its extracellular location within the antennae has been shown by its isolation during the analysis of the soluble proteome of *D. melanogaster *antennae [29]. We show here that *Est-6 *expression in male antennae is high and associated with most of olfactory sensilla, confirming the *Est-6/lacZ *pattern previously observed within the third antennal segment [28]. In addition, we found that *Est-6 *expression is mostly associated with the accessory cells embedding the sensory neurons and located at the base of the sensilla. These cells are already known to produce OBPs and secrete them into the sensillum lymph [35-37]. Altogether, these data suggest that Est-6 could be secreted within the lymph of the olfactory sensilla, including cVA-sensitive sensilla.

As all other β-esterases, Est-6 clustered within a clade that includes extracellular catalytically competent esterases (reviewed in [38]). Phylogenetic analyses [13,39] also revealed that Est-6 was closely related to the antennal carboxylesterases characterized *in vitro *in the wild silk moth *Antheraea polyphemus *[9,12] and in the beetle *Popilia japonica *[8]. These ODEs were able to hydrolyze the female sex pheromones *in vitro *with kinetics suggesting that they could play a significant role in the dynamic of signal termination *in vivo *[8,9,12]. cVA degradation by purified Est-6 into *cis*-vaccenyl alcohol (cVOH) has been shown *in vitro *[24] and it has been also demonstrated that cVOH elicits only very low responses in T1 sensory neurons [40]. Together with its sensillar location, this catalytic activity towards the pheromone suggested that Est-6 could a play a role in pheromone signal termination, as a candidate ODE. Further determination of Est-6 kinetics towards cVA will be useful to precise its mode of action.

If ODEs were required for odorant processing, then their inhibition should disturb odorant reception within the antennae. Several pharmacological approaches have been used to address this question. Volatile trifluoroketones (TFKs), which can inhibit carboxylesterase activities [12] were used in several lepidopteran species to test their effect on pheromone response [10], but controversial effects were observed. In the moth *Ostrinia nubilalis*, prolonged repolarization time of EAG in response to the pheromone after TFK application suggested that esterases were involved in pheromone deactivation [41], but it has also been suggested that TFKs may interact with Ors, OBPs or other members of the transduction cascade [42,43]. Inhibition of antennal cytochrome P450 by metyrapone reduces pheromone responses in a scarab beetle, suggesting that these intracellular enzymes were required for maintaining olfactory sensitivity [11].

In *Drosophila*, genetic tools offer the opportunity to knockdown candidate genes specifically to verify their physiological role directly. This approach led us to demonstrate that the absence of *Est-6 *in males indeed modifies neuronal responses to the pheromone, with stronger and longer-lasting responses. Noteworthy, the kinetics of signal termination within *Est-6° *T1 sensilla is altered even at physiological low doses of pheromone and with brief stimulations, as expected after the knockdown of an ODE. We can assume that the lack of *Est-6 *in mutant antennae prevents the degradation of cVA, which could lead to an accumulation of cVA in the perireceptor space of T1 sensilla. While binding with cVA, the OBP LUSH encounters a conformational change and the LUSH/cVA complex would be the active form that interacts with the receptor (Laughlin *et al.*, 2008). LUSH increases the sensitivity of T1 ORN to cVA but does not to cVOH [21]. In *Est-6° *mutant antennae, accumulation of cVA would thus lead to an accumulation of the complex OBP/pheromone, leading to stronger responses and delayed signal termination. However, Est-6 involvement in signal dynamics does not preclude a role of additional mechanisms in signal termination.

As *Est-6 *has a physiological effect on cVA reception, we thus tested whether *Est-6 *mutation could influence cVA-triggered behaviors. We found that *Est-6 *deficiency clearly enhances the antiaphrodisiac effect of the pheromone. Topical applications of exogenous cVA on females reduced male courtship as already observed [15]. In addition, we showed that the effect of exogenous cVA is dose dependent in control and *Est-6° *males. However, the threshold of behavioral response to the pheromone is lower in *Est-6° *males. Compared to control flies, lower doses of cVA were sufficient to slow down courtship initiation of mutant males, thus to reduce their courtship. *Est-6 *deficiency also increases dispersal thus likely aggression. The proximity to a high density of male flies has been shown to increase the level of male aggression, thus dispersal, in a dose-dependent manner [16]. Dispersal of *Est-6° *males in absence of exogenous cVA suggests again a lower threshold of behavioral response in mutant males.

Activation by cVA of ORNs carrying Or67d in T1 sensilla is sufficient to inhibit male-male courtship behavior [15], and to promote cVA-induced aggression [16]. In particular, increasing artificially the excitability of *Or67d*-expressing ORNs, by expressing a bacterially-derived sodium channel, promotes dispersal of grouped flies even in absence of exogenous cVA [16]. Modified physiological responses of T1 sensilla to cVA in *Est-6° *mutants could thus potentially account for the observed exacerbated behaviors. Delayed cVA termination and stronger responses of T1 sensilla from *Est-6° *mutant males are consistent with their lower threshold of behavioral response. Indirect evidence for a function specific for T1 sensilla comes from the fact that lack of *Est-6 *in mutants did not impair other chemically-driven behaviors, as indicated by a normal response to food odors and to female pheromones.

## Conclusions

In conclusion, we have demonstrated that an extracellular esterase, Est-6, is involved in maintaining proper temporal dynamics of cVA detection at the peripheral olfactory circuit level and is involved in cVA-induced behaviors in males. These results expand the role of Est-6 in *Drosophila *biology, from reproduction to olfaction. After its transfer during mating with the semen, Est-6 is known to rapidly translocate to the female hemolymph and to impact female reproductive behavior (stimulation of egg laying and inhibition of receptivity for remating) [23]. Our results demonstrate that the same enzyme plays a crucial role in cVA detection in male antennae. This work also highlights the physiological role of carboxylesterases in insect odorant reception *in vivo*. In vertebrates, a potential role of extracellular enzymes from the nasal mucus has been recently revealed by a pharmacological inhibition approach [44]. Enzymatic conversion of odorants seemed to be fast enough to affect olfactory dynamics. The corresponding enzymes were not characterized, but carboxylesterases were suspected to play a role in ester conversion [44]. Enzyme-based mechanism of inactivation could be comparable in olfactory systems of insects and vertebrates.

In a context of pest insect management, these enzymes may be interesting targets for the development of specific inhibitors that interfere with the insect's ability to respond adequately to olfactory cues from mates or host plants.

## Methods

### Fly strains, rearing and tissue collection

The following strains were used during this study: an *Est-6° *null mutant strain (Bloomington stock 4211), completely lacking *Est-6 *and described in detail [23]; a rescue strain described in Odgers *et al. *[45], which presents a similar genetic background as *Est-6° *(the *Est-6 *promoter was fused to the *Est-6 *coding region and transformed into the *Est-6 null *background); Canton-S (*CS*) flies were used as wild-type control flies. Transgenic *UAS-mCD8::GFP *(Bloomington stock 5130) flies were used for immunohistochemistry experiments to determine more precisely the expression pattern of *Est-6 *within antennae. For the generation of the *Est-6*^Gal4 ^lines, a 1,132 fragment corresponding to the *Est-6 *promoter region was cloned in the pChs-Gal4 vector, and transgenic flies were generated by P-mediated germline transformation by BestGene Inc. (Chino Hill, CA, USA) according to standard procedures [46]. All flies were raised at 25°C on standard yeast/cornmeal/agar medium in a 12-h light/12-h dark cycle, 50% to 60% relative humidity.

### qPCR

To precisely define the levels of *Est-6 *expression in antennae and other chemosensory appendages, *Est-6 *transcripts were quantified by qPCR analysis. Antennae from 5 to 7-day old males and females, male legs and proboscis with maxillary palps were dissected for total RNA extraction using TRIzol Reagent (Invitrogen, Carlsbad, CA, USA). Heads without appendages were also tested. Single-stranded cDNAs were synthesized from total RNAs (1 μg) using Superscript II reverse transcriptase (Invitrogen). All reactions were performed as previously described [47] on the LightCycler^® ^480 Real-Time PCR System (Roche, Basel, Switzerland). Each reaction was run in triplicate with at least three independent biological replicates. The *pgk*, *rpl8 *and *rp49 *genes were used as reference genes. Specific primers were designed using EPRIMER3 http://mobyle.pasteur.fr and were as follows: *rp49*up CGGATCGATATGCTAAGCTGT, *rp49*do ACGTTGTGCACCAGGAACTT, *rpL8*up TCGTATCGACAAGCCCATCCTGA, *rpl8*do ACCACGGATCCTACCGGTACGAC, *pgk*up CGAGAAACTGGTGGAGAAGG, *pgk*do CGAAGTTGGGGAACTCAAAG, *Est6*up TTCCCGGAAACTATGGACTG and *Est6*do CAGTTCAAAGGCTCGTCCTC. Normalized *Est-6 *expression was calculated with Q-Gene software [48].

### Localization of *Est-6 *expression within antennae

To localize the expression site of *Est-6 *in the antenna, we used transgenic flies expressing GFP under the control of *Est-6 *promoter and we performed immunohistochemistry with an anti-ELAV antibody as neuronal marker. Heads with antennae from 5-day-old males *Est-6^Gal4^/UAS-mCD8-GFP *were fixed for 3 h in 4% paraformaldehyde with 0.2% Triton X-100 (Sigma-Aldrich, Saint-Quentin Fallavier, France), then washed for 1 h with phosphate-buffered saline containing 0.2% Triton X-100 (PBST, Sigma-Aldrich). Heads were then embedded in Tissue-Tek™ (CellPath, Newtown Powys, UK) and cryosections (15 μm) were set in cell culture insert (Greiner Bio-one, Monroe, LA, USA). After blocking with 3% normal goat serum and 1% bovine serum albumin (BSA) in PBST (1 h at room temperature (RT)), an anti-ELAV (from Developmental Studies Hybridoma Bank, University of Iowa) was diluted 1:10 (v/v) in the blocking solution (3% normal goat serum in PBST) and incubated overnight at RT. After a brief rinse in PBST, an anti-mouse conjugated Alexa-546 (Invitrogen) was applied 1:250 (v/v) in the blocking solution for 4 h at RT. tissues were mounted in Slowfade reagent (Invitrogen). Imaging was performed on Olympus BX61 microscope with a ScopePro software.

### Comparison of antennal responses to cVA by EAG

EAG recordings were performed at RT on 5-day-old males previously kept in individual tubes, as described previously [49]. Reference and recording glass capillary electrodes were filled with 120 mM NaCl, 5 mM KCl, 1 mM CaCl_2_, 4 mM MgCl_2_, 10 mM 4-(2-hydroxyethyl)-1-piperazine-ethanesulfonic acid (HEPES, Sigma-Aldrich), pH = 7.2. The reference electrode (approximately 1 μm tip diameter) was inserted in one eye and the recording electrode (approximately 10 μm tip diameter) was pushed against one antenna. The signal was amplified (× 500) and low pass filtered online (10 kHz) with an Axopatch 200B amplifier (Molecular Devices, Union City, CA, USA) and digitized at 1 kHz with a Digidata 1440A acquisition board (Molecular Devices). A dose-response curve was established to select a dose of cVA that induces a high and saturating response. Antennae were then stimulated for 5 s with either pure hexane (> 98% purity, Carlo-Erba Reagents, Val de Reuil, France) as negative control, or with cVA (diluted in hexane, 200 μg/cartridge), or with 2-heptanone (1:1,000 in paraffin oil, > 98% purity, Carlo-Erba) as positive control. Analysis of EAGs was carried out under pClamp 10 (Molecular Devices). Repolarization rates were compared between the different strains. Repolarization rate was defined as: ((maximum amplitude of depolarization - amplitude of depolarization at the end of stimulation) ÷ maximum amplitude of depolarization) × 100.

### Analyses of T1 sensilla responses by single-sensillum recordings

Single-sensillum recordings were performed as described previously [50] in order to follow the response of T1 sensilla more precisely. In brief, a fly was restrained, a reference electrode was placed in the eye, and the recording tungsten electrode was brought in contact with the base of a sensillum. Signal was amplified (× 1,000, Syntech UN 06, Hilversum, The Netherlands). Actions potentials were analyzed offline with Autospike software (v. 4.0, Syntech). Three doses of cVA were tested, 0.5 μg, 5 μg and 50 μg, and two stimulus durations, 0.5 s and 3 s. Responses from individual ORNs were calculated as the increase in spike frequency relative to the prestimulus frequency. Average firing activities during the stimulus duration, as well as during the 9 s period following the end of stimulus were calculated.

### Courtship assays

Males were isolated after emergence and raised in individual tubes to avoid social interactions. All experiments were done under dim red lights at 25°C (50% to 60% relative humidity) and with immobilized target so as to enhance the behavioral effects of pheromone cues [33]. For male-male assays, a single male (5 to 7 days old) was placed in a test chamber (3 cm diameter, 0.5 cm height) for 10 minutes before introducing a decapitated *CS *'target' male. Courtship behavior was observed over 10 minutes and a courtship index (CI) was calculated. CI is the fraction of time spent in courtship activity in the 10-minute observation period. For each tested male, the latency before the first wing vibration and the number of copulation attempts were noted, as an indicator of courtship vigor. Heterosexual courtship was first measured in the same conditions as described for male-male courtship assay, except that a decapitated *CS *'target' virgin female was introduced in the test chamber. Synthetic cVA was then applied on the dorsal abdomen of decapitated virgin females before to offer them to mutant or control males. Mature male flies contain approximately 1 μg of cVA in their ejaculatory bulb [51] and 400 ng on their cuticle [52]. The quantity of cVA transferred to the female during copulation was estimated to 200 ng [24] but 70% of the pheromone is lost 6 h after insemination [17]. The amount of cVA on females 24 h after mating was indeed only 10 ng [32]. Three doses of cVA diluted in acetone (> 98% purity, Sigma-Aldrich) were applied on females, 400 ng (twice male equivalent), 150 ng (approximately two-thirds male equivalent) and 50 ng (one-quarter male equivalent).

### Dispersion assay

Experiments were performed as described previously [16]. Briefly, 15 males were raised together after emergence for 5 to 7 days. Six males were simultaneously introduced in a plastic tube (2.5 cm diameter, 10 cm height) coated with Fluon (Sigma-Aldrich) and a small cup containing food was placed in the center, together with a small piece of filter paper containing either 500 μg of synthetic cVA or acetone (solvent). Flies were videotaped for 30 minutes and the total number of flies on the food cup was counted every 30 s.

### Control of olfactory behavior and of locomotor activity

The response of male flies to airborne chemicals was evaluated using the principle of the olfactory trap assay [34], which allows to test the ability of flies to detect and migrate toward a source of olfactory attractant. All tests were performed under dim red lights as for courtship assays. Ten flies of the same genotype (*CS *or *Est-6° *males) were placed in a 50 ml Greiner tube along with a trap constructed from a microfuge tube and two micropipette tips. Traps contained either a fresh yeast paste included in 10% agar as attractant or only agar. The number of trapped flies was counted after 22 h.

To verify that *Est-6° *males were not impaired in their mobility, their locomotor activity was determined as previously described [53]. In brief, a single male was placed in the test chamber containing a filter paper with a bisecting line. The number of times the male crossed the line in a 3-minute observation period was counted.

### Statistical analysis

Analyses were performed with Statistica 7 (StatSoft Inc., Tulsa, OK, USA). The Student's t test or Mann-Whitney U test were used for pairwise comparisons and analysis of variance (ANOVA) followed by a Fisher post-hoc test was performed for comparisons among multiple groups. *P *< 0.05 was accepted as statistically significant.

## Competing interests

The authors declare that they have no competing interests.

## Authors' contributions

TC and AF carried out electrophysiological, behavioural and molecular analyses, and participated in interpretation of the results and preparation of the manuscript. GR participated in molecular analyses. PL and TD participated in electrophysiological analyses, interpretation of the results and preparation of the manuscript. MMC conceived of the study, participated in interpretation of the results and wrote the paper.

All authors read and approved the final manuscript.

## Supplementary Material

Additional file 1 **Figure S1**. Dose-response curve for *CS *male antennae to *cis*-vaccenyl acetate (cVA), plotted as mean ± SEM; N ≥ 7 for each data point. Click here for file

Additional file 2 **Figure S2**. Control tests for behavioral analysis. **(A) **Olfactory trap assay using fresh yeast paste as attractant. **(B) **Locomotor activity. Mean ± SEM; N = 10 and 40, respectively. Student's t test, *P < 0.05. Click here for file
