# Supplementary figures and images for: A carboxylesterase, Esterase-6, modulates sensory physiological and behavioral response dynamics to pheromone in Drosophila
Source: BMC Biol. 2012 Jun 21;10:56. doi: 10.1186/1741-7007-10-56 (PMC3414785; doi:10.1186/1741-7007-10-56)

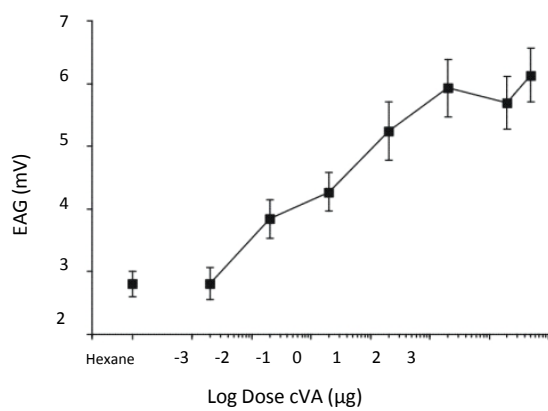

Supplement: Additional file 1 — Figure S1. Dose-response curve for CS male antennae to cis-vaccenyl acetate (cVA), plotted as mean ± SEM; N ≥ 7 for each data point. [file 1741-7007-10-56-S1.PDF]

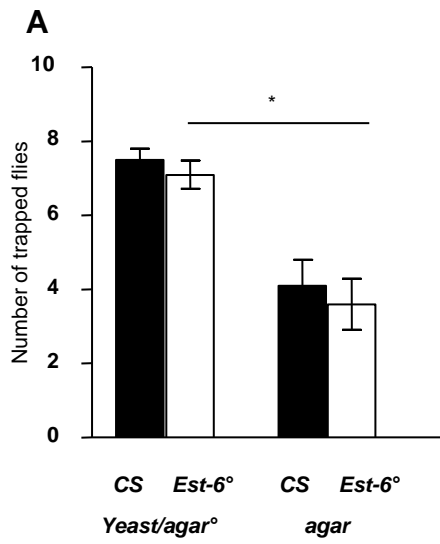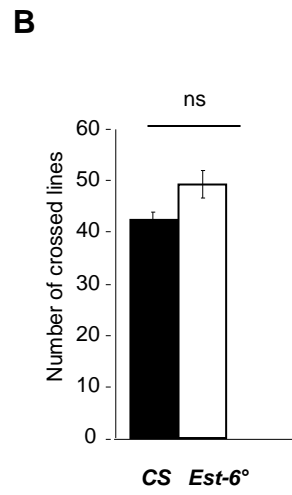

Supplement: Additional file 2 — Figure S2. Control tests for behavioral analysis. (A) Olfactory trap assay using fresh yeast paste as attractant. (B) Locomotor activity. Mean ± SEM; N = 10 and 40, respectively. Student's t test, *P < 0.05. [file 1741-7007-10-56-S2.PDF]
